# Breastfeeding and Immunohistochemical Expression of ki-67, p53 and BCL2 in Infiltrating Lobular Breast Carcinoma

**DOI:** 10.1371/journal.pone.0151093

**Published:** 2016-03-10

**Authors:** Angel Gonzalez-Sistal, Alicia Baltasar-Sánchez, Primitiva Menéndez, Jose Ignacio Arias, Álvaro Ruibal

**Affiliations:** 1 Department of Physiological Sciences II, Faculty of Medicine, University of Barcelona, Barcelona, Spain; 2 Pathology Service, Hospital Central de Asturias, Oviedo, Spain; 3 Surgery Service, Hospital Monte del Naranco, Oviedo, Spain; 4 Nuclear Medicine Service, Complejo Hospitalario Universitario, Faculty of Medicine, IDIS, Santiago de Compostela, Fundación Tejerina, Madrid, Spain; Queen Mary Hospital, HONG KONG

## Abstract

**Background/Aim:**

Invasive lobular breast carcinoma is the second most common type of breast cancer after invasive ductal carcinoma. According to the American Cancer Society, more than 180,000 women in the United States find out they have invasive breast cancer each year. Personal history of breast cancer and certain changes in the breast are correlated with an increased breast cancer risk. The aim of this work was to analyze breastfeeding in patients with infiltrating lobular breast carcinoma, in relation with: 1) clinicopathological parameters, 2) hormonal receptors and 3) tissue-based tumor markers

**Materials and Methods:**

The study included 80 women with ILC, 46 of which had breastfeed their children. Analyzed parameters were: age, tumor size, axillary lymph node (N), distant metastasis (M), histological grade (HG), estrogen receptor (ER), progesterone receptor (PR), androgen receptor (AR), Ki-67, p53 and BCL2

**Results:**

ILC of non-lactating women showed a larger (p = 0.009), lymph node involvement (p = 0.051) and distant metastasis (p = 0.060). They were also more proliferative tumors measured by Ki-67 (p = 0.053). Breastfeeding history did not influence the subsequent behavior of the tumor regardless of histological subtype

**Conclusion:**

Lactation seems to influence the biological characteristics of ILC defining a subgroup with more tumor size, axillary lymph node involvement, distant metastasis and higher proliferation measured by ki-67 expression.

## Introduction

Invasive (or infiltrating) lobular breast carcinoma (ILC) starts in the milk-producing glands (lobules). It is the second most common type of breast cancer after invasive ductal carcinoma (cancer that begins in the milk-carrying ducts and spreads beyond it). According to the American Cancer Society, more than 180,000 women in the United States find out they have invasive breast cancer each year. ILC can spread (metastasize) to other parts of the body. About 10% of all invasive breast cancers are invasive lobular carcinomas. ILC may be harder to detect by a mammogram than invasive ductal carcinoma because it typically doesn't form a lump, which is common in breast cancer. Instead, there is a change in the breast that feels like a thickening or fullness in one part of the breast and is different from the surrounding breast tissue.

Lactation is attributed with a range of relative risk reductions, ranging from 4.3–6.4%. We know is a lower risk factor [1–2], mainly from hormone-dependent tumors [3], both invasive and in situ adenocarcinoma subtype and the risk decreased for each 12 months of lactation [4]. According to current knowledge, it is important that lactation appears to mainly reduce the risk of basal cell carcinomas/triple negative [5–6], some authors extend this to luminal [7]. Among the pathophysiological mechanisms of lactation we can found anovulation, cellular differentiation of mammary cells and milk carcinogens excretion. After treatment of breast cancer there is no evidence that lactation increases the risk of recurrence [8]. The main prognostic factors associated with breast cancer are the number of lymph nodes involved, tumor size, histological grade, and hormone receptor status, the first two of which are the basis for the AJCC staging system. However, after determining the stage, histological grade, and hormone receptor status, the tumor can behave in an unexpected manner, and the prognosis can vary. Other prognostic and predictive factors have been studied in an effort to explain this phenomenon, some of which are more relevant than others: Ki-67, p53, BCL2 [9].

The aim of this work was to analyze breastfeeding in patients with lobular breast carcinoma, in relation with: 1) clinicopathological parameters, 2) hormonal receptors and 3) tissue-based tumor markers.

## Materials and Methods

### Patients

80 women affected by breast ILC (other histological subtypes were excluded), of which 46 had breastfed their children. Women were aged between 30 and 87 years (mean age 58.7±10.4l) and were studied at Breast Unit at the Monte del Naranco Hospital, Oviedo, Spain. They were selected from a breast cancer screening program from 2000 to 2007, written informed consent was obtained from all participants, and the study was approved by the Institutional Review Board of Universitat de Barcelona (IRB 00003099).

### Methods

Given the heterogeneity in time and number of children, lactating women have considered only those that were lactating at least eight months [10], regardless of the number of children. Parameters analyzed were: age, tumor size, axillary lymph node (N), distant metastasis (M) and histological grade (HG). We also considered immunohistochemical expression of estrogen receptor (ER), progesterone receptor (PR), androgen receptor (AR), BCL2, p53 and Ki-67.

Immunohistochemical staining on tissue sections of 4–5 microns was performed by *EnVision* method with a heat-induced antigen retrieval step. Sections were immersed in boiling 10 mmol/l sodium citrate at pH 6.5 for 2 min in a pressure cooker. ER and PR were determined using monoclonal antibodies to ER and PR phramDx (clones 1D5 and ER-2123, respectively), 1294 for the PR, p53 (DO-7, dilution 1/ 50; Dako), Ki-67 (MIB-1, dilute 1/ 200; Dako), BCL2 (Biogenex, dilution 1/ 150) and androgen receptor (AR441, dilution 1/ 150; Dako) were used in this study. ER and PR were assessed according to the Allred score [11] as negative (scores 0–2) and positive (score 3–8), and positivity thresholds for p53 and Ki-67 were 20% and 15%, respectively [12]. AR was classified as positive or negative without any score, and BCL2 as negative (-), weakly positive (+) and strongly positive (++).

The Windows SPSS software was employed for statistical analysis (SPSS, Chicago, IL, USA). Continuous variables with a normal Gaussian distribution are expressed as the mean and standard deviation. We used the Chi-square test with Yates correction, if necessary, for comparison of qualitative variables, and Mann Whitney test for continuous ones. The criteria to be considered significant was as *p*<0.05.

## Results

In the study group patients were aged between 30 and 87 years. Pathological tumor size ranged from 0.3 and 10cm. Results were divided into two groups: breastfeeding and no breastfeeding.

Table 1 shows the relationship between lactation and clinicopathological parameters in women with breast ILC. Women without previous breastfeeding have larger tumor size ranged between 0.9 to 10 cm. (p = 0.009), lymph node involvement (p = 0.051) and distant metastasis (p = 0.060).

**Table 1:** Relationship between breastfeeding and clinicopathological parameters in patients with infiltrating lobular breast carcinoma (ILC). A p-value of 0.05 was considered to be significant.

	n	Breastfeeding	n	No Breastfeeding	*p*-Value
**Age**	46	42–87 (59.1±11.3)	34	30–82 (58.0±12.7)	ns
**Size**	46	0.3–6.5 (1.9±1.4)	34	0.9–10 (3.3± 2.2)	0.009
**N**		13/46		19/34	0.051
**N>3**		4/46		8/34	ns
**M**		2/46		6/34	0.06
**HG3**		4/36		4/32	ns

N: lymph node; M: distant metastases; HG: Histological Grade

Table 2 shows lactation according to the hormonal receptors and tissue-based tumor markers analyzed. There were no significant differences when the expression of ER, PR and AR was considered. We found statistically significant differences in women without previous breastfeeding have more proliferative tumors measured by Ki-67 expression (p = 0.053).

**Table 2:** Relationship between breastfeeding, hormonal receptors and tissue-based tumor markers in patients with infiltrating lobular breast carcinoma (ILC). A p-value of 0.05 was considered to be significant.

	Breastfeeding	No Breastfeeding	*p*-Value
**ER+**	36/36	25/28	ns
**PR+**	23/35	20/28	ns
**AR+**	22/27	25/25	ns
**Ki-67 +**	8/35	16/28	0.053
**P53 +**	2/28	6/27	ns
**BCL2 ++**	21/28	20/24	ns

ER: estrogen receptor; PR: progesterone receptor; AR: androgen receptor

## Discussion

Worldwide, more women develop breast cancer than any other malignancy. Invasive ductal and lobular breast carcinoma, constitute the largest group of breast tumours, comprising up to 95%of all breast cancer. The interactions between pregnancy and breast cancer are complex and variable. The influence of pregnancy on the risk of developing breast cancer is dependent on maternal features. The risks related to pregnancy history are not currently incorporated into clinical tools for assessing woman’s risk for the development of breast cancer. Some studies have suggested that breastfeeding reduces breast cancer risk, but evidence has been mixed.

In the present study, we analyzed possible associations between lactation and clinicopathological factors commonly used in daily clinical practice in patients with breast ILC. We found absence of lactation was associated with larger tumors, more axillary lymph node involvement and distant metastases, which reflect a poorer outcome. Several hypotheses explain the protective effect of lactation. First, lactation promotes differentiation of mammary epithelial cells less susceptible to carcinogenic stimuli, rendering them less susceptible to carcinogenic stimuli [13]. Second, length of lactation further decreases a woman’s lifetime exposure to cycling hormones over pregnancy alone by further suppression of ovulation [14]. Third, recently studies indicate that the lactation environment is tumor protective in rodents [15–16].

We found a statistically significant association between ki-67 expression and women without previous breastfeeding. There was also more proliferation measured by immunohistochemical expression of Ki-67. About Ki-67, we know that is a factor of poor prognosis in early breast cancer patients treated with radiotherapy and breast conservation [17], that in patients with breast cancer without axillary lymph node it is an independent prognostic factor in the 87% of the patients who had not received adjuvant medical treatment. Highlight that prognostic information of Ki-67 is restricted to ER-positive patients with histological grade 2 [18–20]. Ki-67, as an easily assessed and reproducible proliferation factor, may be complement to histological grade as a prognostic tool for selection of adjuvant and treatment, which is a robust cost-effective diagnostic tool that subdivides grade 2 carcinomas into low and high risk populations providing additional prognostic information in planning and outcome prediction therapies [21]. In the same way, proliferation study has acquired great value with the new molecular classification of breast tumors, and some authors consider necessary to change the guidelines and to include Ki-67 in the standard pathological assessment of early breast cancer [22].

Our preliminary results, based on the limited number of cases included in the study, led us to the following consideration: lactation seems to influence the biological characteristics of ILC defining a subgroup with more tumor size, axillary lymph node involvement, distant metastasis and higher proliferation measured by ki-67 expression.

## Abbreviations

ILC: infiltrating lobular breast carcinoma
N: lymph node
M: distant metastases
ER: estrogen receptor
PR: progesterone receptor
AR: androgen receptor
HG: histological grade
